# Preliminary studies of cell culture strategies for bioprocess development based on HEK293 cells

**DOI:** 10.1186/1753-6561-7-S6-P52

**Published:** 2013-12-04

**Authors:** Leticia Liste-Calleja, Jonatan López-Repullo, Martí Lecina, Jordi Joan Cairó

**Affiliations:** 1 Chemical Engineering Department, Universitat Autònoma de Barcelona, Cerdanyola del Vallès, 08193, Spain

## Background

The use of human embryonic kidney cells (HEK293) for recombinant protein or virus production has gained relevance along the last years. They are specially recommended for transient gene expression and adenovirus or adeno-associated virus generation [1,2]. To achieve high volumetric productivities towards bioprocess optimization, the concentration of biocatalizer (i.e. animal cells) must be enhanced. The limits for cell growth are mainly related to the accumulation of metabolic by-products, or the depletion of nutrients [3]; therefore, cell cultures strategies must be developed. In this work, we have explored Punctual Feeding and Media Replacement cell culture strategies to over perform the limit on Xv_max _encountered on batch culture mode. Finally, we scaled up cell culture in order to control other parameters (i.e. pO_2_) that could be limiting cell growth.

## Materials and methods

The cell line used in this study was HEK293SF-3F6 (kindly provided by Dr. A.Kamen, NRC-BRI). The basal medium for all cell cultures was SFMTransFx-293 (Hyclone, Thermo Scientific) supplemented with 5% (v/v) of FBS and 4 mM GlutaMAX (Gibco, Invitrogen). For Punctual Feeding and FedBatch Fementation Cell Boost 5 (Hyclone, Thermo Scientific) was used. Batch, media replacement and punctual feeding experiments were performed in 125-ml plastic shake flasks (Corning Inc.) shaken at 110 rpm in an orbital shaker at 37°C, 95% humidity, 5% CO_2 _incubator. FedBatch Fermentation was carried out in Bioreactor Braun-MCD (2 L) with mechanical agitation at 80 rpm, pH set point 7.1 and pO_2 _set point 50%. Viable cell density and viability were determined by trypan blue exclusion method and manual counting using a haemocytometer. Glucose and lactate were analysed in an automatic analyser, YSI (Yellow Springs Instrument, 2700 Select).

## Results

Characterization of HEK293 cell culture in batch operation was initially performed. It was encountered that cell growth was extended for 168 h reaching approximately 7·10^6 ^cell/mL of cell density (Figure 1.1). Nevertheless, maximal cell growth rate (μ_max_) was only maintained for 96 h. As glucose and lactate were not at limiting concentrations [4], nutrient limitation different from glucose arose as the first hypothesis for this decrease on cell growth rate. Therefore, punctual additions of nutritional supplement for HEK293 were carried out. Xv_max _was significantly increased in comparison to basal media, reaching cell densities as high as 17·10^6 ^cell/mL (Figure 1.2). Nevertheless, we could not overcome this limit on Xv_max _regardless the number of punctual feedings performed. Moreover, nutrient addition did not elongate μ_max _period (t_μ_). These results suggested that by-product accumulation different from lactate could be limiting cell growth. In order to validate the hypothesis, complete media replacement (up to three replacements) was studied (Figure 1.3). Although this strategy enabled to extend t_μ _up to 168 h of cell culture, the maximal cell density reached was similar to nutrient addition strategy (1MR: 12·10^6 ^cell/mL; 2MR: 16·10^6 ^cell/mL; 3MR: 18·10^6 ^cell/mL). This limit on Xv_max _encountered on shake flask might be related to a limitation on pO_2. _Thus, the cell culture system was changed towards a bioreactor with controlled pO_2 _(maintained between 20-60% of air saturation). In addition, a continuous feeding using a pre-fixed profile addition was implemented. As it can be noticed in Figure 1.4, FedBatch operation in bioreactor enabled to beat the limit encountered in shake flask system, reaching cell densities of 27·10^6 ^cell/mL.

**Figure 1:**
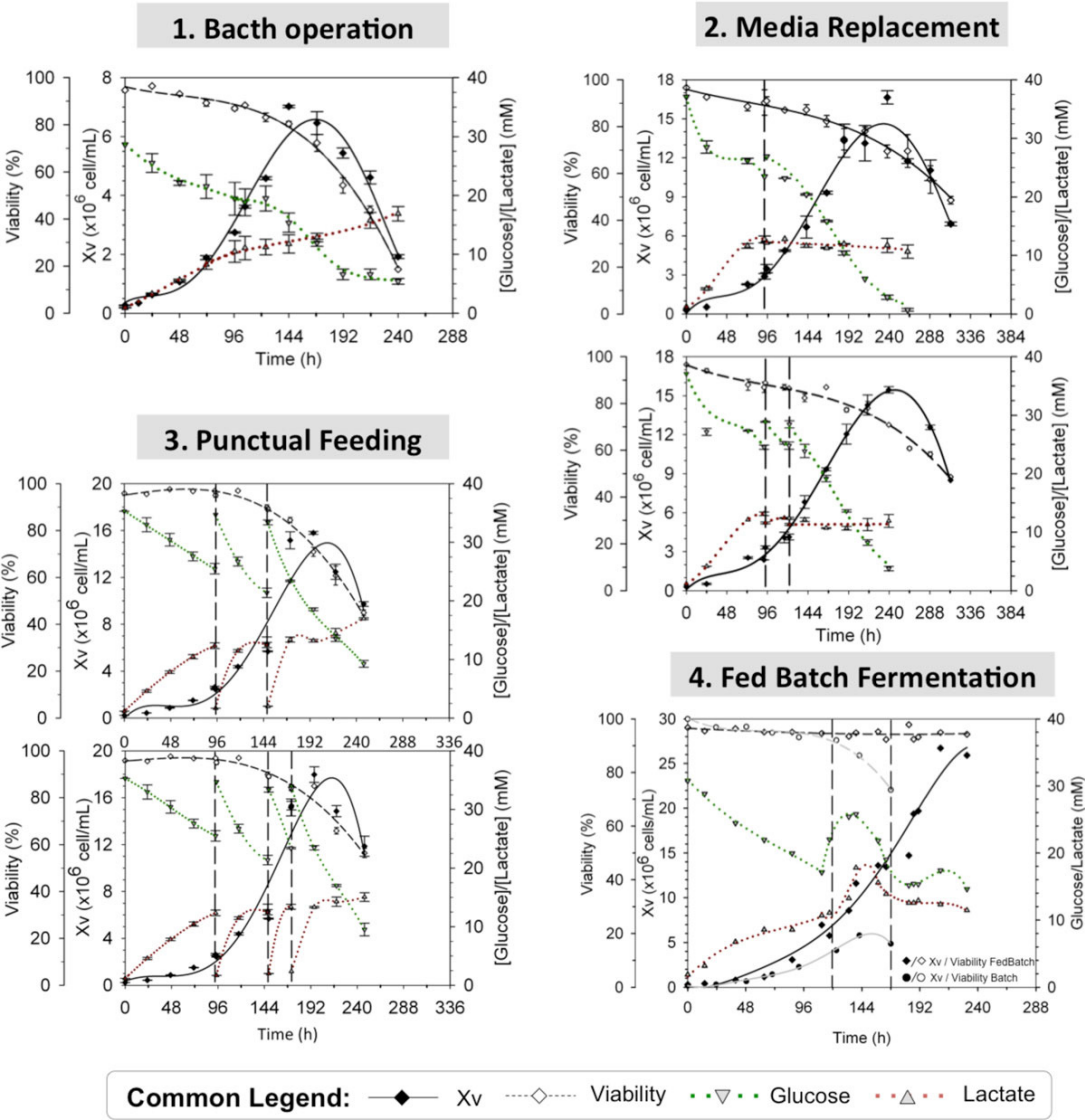
**Comparison of HEK293 cell growth, viability, glucose and lactate profiles in different cell culture strategies**.

## Conclusions

Punctual feeding and media replacement overcame the limit of 7·10^6 ^cell/mL encountered in batch mode operation indicating that nutrient depletion was one of the causes of that limit. Nevertheless, the elongation of t_μ _found out performing MR suggests that the accumulation of by-products might not be ruled out.

The new limit on Xv_max _(≈17-18·10^6 ^cell/mL) encountered regardless the cell culture strategy, was outperformed by transferring O_2 _more efficiently in bioreactor system, reaching cell densities as high as Xv_max _= 27·10^6 ^cell/mL. The monitoring and control of cell culture parameters (i.e. pO_2_, pH) will enable to develop more accurate feeding strategies in order to achieve higher cell densities than those presented here (on going work).
